# How to conduct good quality research on violence against children with disabilities: key ethical, measurement, and research principles

**DOI:** 10.1186/s12889-019-7456-z

**Published:** 2019-08-17

**Authors:** Nambusi Kyegombe, Lena Morgon Banks, Susan Kelly, Hannah Kuper, Karen M. Devries

**Affiliations:** 1 Gender Violence and Health Centre, Department of Global Health and Development, London School of Hygiene and Tropical Medicine, 15-17 Tavistock Place, 15, London, WC1H 9SH UK; 2 International Centre for Evidence in Disability, Department of Clinical Research, London School of Hygiene and Tropical Medicine, Keppel St, London, WC1E 7HT UK

**Keywords:** Violence against children, Disability, Research ethics, Child protection, Low and middle-income settings

## Abstract

**Background:**

Approximately one billion children experience violence every year. Violence against children is an urgent global public health concern and violation of children’s rights. It is also a risk factor for serious negative health and social outcomes and is therefore addressed within the Sustainable Development Goals (SDGs). Children with disabilities, who make up one in 20 children worldwide, are particularly vulnerable to violence although good quality data are lacking on causes and means of prevention of violence against children with disabilities. Key challenges exist in the measurement of disability and violence, which in part explains the dearth in evidence.

**Improving research on violence against children with disabilities:**

This paper provides guidance on how to conduct good quality, ethical, and inclusive research on violence against children with disabilities, particularly in low-income settings. The lack of an international agreed ‘gold standard’ frustrates efforts to measure violence across settings and time. Careful consideration must be given to the design of survey tools. Qualitative and participatory research methods also offer important opportunities to explore children’s subjective understanding and experiences of violence. Challenges also exist around the measurement of disability. Disability may be measured by asking directly about disability, through self-reported functioning, or through the presence of impairments or health conditions. These approaches have strengths and limitations and should build on what children are able to do and include appropriate adaptations for specific impairments where necessary. Ethical research also requires adherence to ethical guidelines and approvals, obtaining informed consent, appropriate child protection responses, and careful consideration of interviewer-related issues including their selection, training, and welfare. Key methodological gaps remain - how to include children with severe communication challenges in research; how to respond in instances of weak child protection systems; designing sampling procedures that adequately represent children with disabilities in large-scale violence surveys; and determining how best to ask about violence safely in large-scale surveys and monitoring data. This paper further advocates for the dissemination of research results in inclusive and accessible formats.

**Conclusion:**

With careful planning, challenges in collecting data on disability and violence can be overcome to generate evidence in this neglected area.

## Background

An estimated one billion children, half of all the world’s children, experience violence every year [1, 2]. This violence can take a number of forms including neglect; physical, emotional or sexual abuse [3]; economic violence; and physical and sexual exploitation. Violence against children is a global public health concern and a violation of children’s rights [4]. Violence in childhood is also a known risk factor for serious negative health and social outcomes. These include anxiety and depression [5–7], poorer health status in adulthood [8], increased risk of victimisation (for girls) or perpetration (for boys) of interpersonal violence in later life [8, 9], and drug and alcohol misuse [10, 11]. They also include poor educational outcomes [12, 13], externalising and conduct disorders [5, 6, 12, 14, 15], delinquency and criminal behaviour, negative interpersonal conflict resolution [16], and risky sexual behaviour [10].

The importance of reducing violence perpetrated against children is increasingly recognised, including in the Sustainable Development Goals (SDGs), adopted in 2015. These goals emphasise: the importance of ending all forms of violence against children (included under Goal 16); the need to achieve gender equality and the elimination of all forms of violence against women and girls (included under Goal 5); and the importance of ensuring that education facilities are child, disability, and gender sensitive, and provide safe, non-violent and effective learning environments for all (included under Goal 4) [17]. An overarching aim of the SDGs is to “leave no one behind”, with specific reference to inclusion of people with disabilities. Consequently, in order for the SDGs to be successfully achieved, efforts to meet the SDGs need to be equitable, and inclusive of all children, including those with disabilities.

Certain groups of children are known to be particularly vulnerable to violence, and this includes children with disabilities [18, 19]. Childhood disability is common – about one in 20 children have a disability worldwide [20]. Globally, UNICEF estimates that up to 150 million children aged 0–18 years live with a disability [3]. This figure may be higher still as the World Health Organization estimates that up to one in five children and adolescents experiences a disabling mental illness [21] with other studies also suggesting similar prevalence of children living with disabilities [22]. Existing research highlights that both adults and children with disabilities are more vulnerable to violence, although most of the data comes from high-income countries [19, 23]. For instance, a recent systematic review of 17 studies from high-income countries found that children with disabilities were three to four times more likely to experience violence compared to their peers without disabilities [19].

The limited data that is available from low- and middle-income countries (LMICs) shows that violence against children with disabilities is also a concern in those settings. As an example, a recent study from Uganda showed that among all children attending primary school, 95.5% of girls without a disability and 99.5% of girls with a disability (*p* = 0.0009), and 95.3% of boys without a disability and 96.2% of boys with a disability (*p* = 0.644) had experienced violence in their life time [18]. The reasons why children with disabilities are more vulnerable to violence are also poorly understood. Violence may be linked to negative attitudes and misconceptions about disability, as well as barriers to participation in society [24]. Children with disabilities are also more likely to experience poor health [25–27], live in poverty [28] and be excluded from education [29, 30]. These exclusions may exacerbate vulnerability to violence and may make it more difficult for children with disabilities to access child protection. There is also a dearth of evidence on which interventions may be effective to prevent violence against people with disabilities [31].

The risks associated with not doing research on children’s experiences of violence should be acknowledged. Research is not only essential to the advancement of health and well-being, but the participation of children and young people in research is also critical for ensuring that their experiences and perspectives are responsibly represented in policy and interventions that affect them to ensure that they receive the full benefits of research [32]. Efforts to understand, prevent and respond to violence against children, including those with disabilities, rely to a large extent on having access to good quality data, including on its magnitude, nature and consequences. This evidence is currently lacking [19], although an increasing number of governmental, non-governmental and civil society organisations are asking children about their experiences of violence [33]. The Centers for Disease Control’s Violence Against Children Surveys (VACS), for example, seek to provide nationally representative data on physical, emotional and sexual violence against girls and boys [34] although these surveys exclude children with mental disabilities which prevent them from being able to understand the questions asked, or physical impairments (such as hearing and speech impairments) that prevent an interviewer from administering the survey. While there are several challenges to the participation of children with disabilities in violence-related research, there is an inherent need to balance the risk of silencing or excluding children with disabilities (and thus not hearing or even misrepresenting their voices) in the name of protecting them, with the risk of coercing or exploiting their involvement [35]. Research must be inclusive of children with disabilities and as such, more effort is needed to overcome the barriers that many children and young people with disabilities face in participating, as is their right [36, 37]. Studies might benefit from the inclusion of measures of disability within the large surveys used to measure violence and monitor progress on the SDGs (for example VACS surveys, WHO’s Global School-based Student Health Surveys, and Health Behaviour in School aged children surveys). Furthermore, it will likely also require new approaches to be used to ensure that the voices of children with disabilities are included.

There is also a need for inclusive qualitative research on violence against children to understand better children’s subjective experiences, voices and perceptions of violence, as well as the particular vulnerabilities that children with disabilities face with relation to violence. These efforts will require researchers to adapt their methods in order to involve children with disabilities in research, for instance considering new ways to communicate. This paper draws on the authors’ experience of conducting research on violence against children in low- and- middle-income settings and seeks to highlight key ethical, measurement and research principles that should be considered when conducting good quality research on violence against children with disabilities in order to help fill these evidence gaps.

### Measurment of violence and disability

An important first step when conducting research on violence against children with disabilities is determining how to measure and define both of these concepts.

#### Measuring violence

Violence against children can take a number of forms including neglect and physical, sexual, and emotional abuse, economic violence, and exploitation. While many organisations and individuals are active in research on violence against children, the lack of an internationally agreed ‘gold standard’ on how to define and measure this sensitive issue, has resulted in the development of diverse indicators, questionnaires and study designs [38, 39]. This diversity makes comparability of data across contexts and time difficult, with implications on how to monitor the impact of efforts to prevent violence against children [38, 40].

Both quantitative and qualitative measures of violence are important. In measuring the prevalence of violence, international surveys tend to ask about specific acts of violence, for example asking a respondent if they have been “slapped”, “punched”, or “kicked”, rather than asking if they have “ever experienced violence”. This is because violence may be defined differently in different contexts, with some violent acts not necessarily being construed as ‘violence’ in some settings. A number of institutions have attempted to establish internationally validated tools for researching violence against children. These include the Centre for Disease Control’s Violence Against Children (VACS) surveys [2]; the International Society for the Prevention of Child Abuse and Neglect (ISPCAN) Child Abuse Screening tools (ICAST) [41]; the Juvenile Victimisation Questionnaire (JVQ) [42]; modules from UNICEF’s Multiple Indictor Cluster Surveys (MICS) [43] as well as questions that are included in school-based surveys including the Health Behaviour in School-aged Children (HBSC) [44].

Qualitative methods also offer important opportunities to explore children’s subjective experience and understanding of violence and its effect on them and their lives. Through these methods, including in-depth interviews and participatory methods, children can be supported to discuss their experiences in a way, and at a pace, that is comfortable for them. Going beyond pre-determined survey items, qualitative methods also provide a means to explore unanticipated aspects of children’s experience of violence while allowing children to discuss their experiences in their own words. Similarly, the use of ‘body maps’ (graphical representations of child’s body), ‘feeling dice’ (dice with different feelings on each face such as happiness, anger, fear, surprise, hurt and sadness) and ‘story boards’ (pictorial representations of different physical spaces in a child’s immediate environment) provide participatory tools to help children talk about where on their body they experience violence, how this violence makes them feel, and where in their physical environment violence takes place. These methods may help researchers and programmers to better understand how violence affects children as well as specific places, circumstance and locations in which children are exposed to violence, so that appropriate interventions can be developed.

In developing both quantitative and qualitative tools designed to measure violence, researchers should pay careful attention to the structure of the tools to ensure that sensitive questions (e.g. about sexual violence) are asked neither at the beginning nor the end of the interview. This enables interviewers to build a rapport and trust with study participants before asking sensitive questions, and also helps interviews to end on a positive note. Given that carers also form an important part of the support system for children with disabilities, it is also important to build social trust with carers, to highlight positive aspects of carer-child relationships [45] and to take full and meaningful account of children’s knowledge and views [46]. On the completion of the interview, research participants should be asked about how they feel about having participated in the study. This can be done either verbally or through using age and culturally appropriate written methods such as asking the child whether their feelings are best represented by a smiley face or a sad face. At the completion of the interview, interviewers should also remind participants of the availability of referrals for example for psychosocial or child protective services should they wish to access them or should the interviewer consider that they would benefit from being referred (this is discussed in more detail below).

#### Measuring disability

There are also challenges around the conceptualisation and measurement of disability. Disability is a complex umbrella term with many culturally constructed meanings. A frequently used definition of disability is that given by the United Nations Convention of the Rights of Persons with Disabilities, as “long-term physical, mental, intellectual or sensory impairments which, in interaction with various barriers, may hinder [a person’s] full and effective participation in society on an equal basis with others”. Essentially, this means that children with disabilities are those who have an impairment (e.g. visual, hearing, intellectual, psychosocial, physical) which in combination with personal and environmental barriers (e.g. inaccessible buildings, stigma and negative attitudes, lack of assistive devices) restricts their full participation (e.g. in school, social life). Just as there is complexity about how we define disability, there is also complexity on how we measure disability and the words that we use to describe disability. Indeed, in many contexts there are no single words that describe disability, and many of the words used have negative and stigmatising connotations.

The simplest method for measuring disability is through a binary question on whether the respondent considers him/herself to have a disability. While this approach has been used historically to measure disability, it is not recommended as it is increasingly recognised to severely underestimate the prevalence of disability [47], for example where people do not want to identify as having a disability, for fear of stigma or discrimination.

Disability has also been measured in past research through clinical assessments of impairments (e.g. measuring visual acuity for visual impairments, determining the presence of medical conditions and impairments). While this approach can provide useful information, particularly in planning medical services, it is not recommended in isolation for determining disability. Impairments may not be an accurate proxy for disability if assessments do not take into consideration the impact of the impairment on performing daily activities or social participation. For example, two individuals may both have the same level of visual impairment, but if one wears glasses and one does not, the disabling impact of visual impairment on the individual without glasses will be much greater. Further, the extent to which societies are organised to support the inclusion of people with diverse impairments (e.g. social norms and attitudes, accessibility of physical environments, information) plays a large role in mitigating or exacerbating experiences of disability arising from an impairment. Consequently, clinical assessments do not capture important elements of disability as defined by the United Nations Convention on the Rights of Persons with Disabilities (UNCRPD). Clinical assessments also tend to be resource intensive and often require specialised staff, both of which may be in short supply in certain contexts.

A third method for determining the presence of disability is through assessments of functioning – either self-reported or objectively measured. These methods have increasing been used to measure disability and are more in line with dominant conceptualisations of disability espoused in the UNCRPD and the World Health Organisation’s International Classification of Functioning, Disability and Health (ICF) than the other two approaches described [48, 49]. Functioning-based assessments focus on difficulties in performing different daily life activities (e.g. seeing, hearing, walking, understanding) in a given context, with or without the presence of supports (e.g. glasses, hearing aid, walker). Functioning-based approaches are used by the United Nation’s Washington Group on Disability Statistics who, in partnership with UNICEF, have developed the Child Functioning Modules [50]. The Chid Functioning Modules are recommended for data collection on disability amongst children aged 2–17 years by global stakeholders, including the United Nations Statistics Division [51–53]. This question set has been validated and used for research in a range of contexts, and official translations are available in ten languages [54]. A key aim of these question sets is to promote international comparability in the assessment of childhood disability, and to capture important aspects of child functioning and development. Measuring disability with the Child Functioning Modules can therefore be used to embed research findings within a growing evidence base on childhood disability.

The Childhood Functioning Module contains two question sets: one for children aged five to 17 years and another for children 2–4 years old [52, 55, 56]. The modules contain 16 to 24 questions, which cover difficulties experienced performing the following activities:

- Children 5–17 years: seeing, hearing, walking, self-care, being understood, learning, remembering, concentrating, accepting changes in routine, controlling their behaviour, making friends, anxiety, depression
- Children 2–4 years: seeing, hearing, walking, picking up objects, understanding, being understood, learning, playing with others, controlling behaviour

Typically, primary caregivers report on the functioning of their children, although for adolescents, self-reporting has sometimes been used [51, 57]. All questions ask caregivers to compare the child’s functioning to other children of the same age and while using available assistive devices (e.g. glasses, hearing aids, walking equipment). For most questions, four response options are available: no difficulty, some difficulty, a lot of difficulty or cannot do. For controlling behaviour in children 2–4 years, options are: not at all, the same or less, more or a lot more. For anxiety and depression, response options centre on frequency: daily, weekly, monthly or never. Although various cut-offs have been used, the following are recommended by the Washington Group-UNICEF for measurement of disability [51]:

- Children 5–17 years: “a lot of difficulty” or “cannot do” for at least one domain or “daily” anxiety/depression
- Children 2–4 years: “a lot of difficulty” or “cannot do” for at least one domain or “a lot more” for controlling behaviour

The Washington Group Short Set may be used in lieu of the Child Functioning Modules, as it is shorter in length (six questions) and may be used across adults and children (ages 5+) [54, 58]. The Short Set may be more appropriate for censuses or all-age, multi-topic surveys; however, it is acknowledged that it does not capture the complete range of functional domains that are important for child development [51].

### Ethical issues

#### Ethical guidelines and approvals

Institutional Review Boards (IRB) may sometimes be hesitant to approve trauma-related research involving children for fear that this will result in the re-victimisation of survivors of violence [59], especially those who are considered particularly vulnerable such as children with disabilities. As discussed above, it is important to balance the risks of not including children with disabilities in research that affects them, with the potential positive outcomes as a result of the research in terms of the development of appropriate interventions. Increasingly, there is also evidence to show that while being asked about traumatic experiences may cause some upset particularly for younger children, on balance, inquiries into prior episodes of childhood victimisation are generally well tolerated by children, and adults who experienced violence in childhood [59–61].

A number of ethical guidelines exist which provide guidance on conducting research involving children [62–65], with most requiring extra protection for vulnerable groups, such as children with disabilities. These guidelines help to minimise the risk of potential harm resulting from data collection to participants, researchers and others while helping to ensure that any remaining risks are outweighed by potential benefits. For children, particularly those with disabilities, researchers must ensure that participation in data collection does not do any harm, particularly if disclosing abuse puts them at increased risk of abuse from the perpetrators of the violence. The requirement to do no harm must also endure even after the end of the study.

To ensure ethical oversight, research protocols, procedures, tools and consenting procedures must first be submitted for approval through appropriate in-country and international ethical review boards, with all procedures and recommendations adhered to when conducting research. Given that not all ethics review boards will have experience of research involving violence against children, or children with disabilities, researchers must hold themselves accountable to the highest standards of ethical research with specific attention to international guidelines on research involving children and research on violence [33, 63, 65]. Ethical considerations will be the same as for most other studies, such as confidentiality of information and protection from harm. However, there are two particular areas where additional attention is required when collecting data on violence from people with disabilities – the first is informed consent and the second is the implementation of appropriate child protection responses.

#### Informed consent and assent

The importance of obtaining informed consent for participation in research cannot be overstated given the sensitivity of violence research, and the potential vulnerability of children in general, and those with disabilities in particular [35, 36]. Before commencing research therefore, basic informed consenting procedures require that interviewers explain to the child what participation will involve and that participation is voluntary, the potential risks and benefits of participation, and their rights to withdraw from the study or not answer any question. Researcher should also ensure that practical measures are taken so that participants do not experience any negative consequences as a result of declining to participate or due to their responses. For example, if recruitment of participants is conducted through Disabled Persons’ Organisations (DPOs) or service providers, it must be made explicitly clear that decisions to participate and responses given will not in any way affect the ongoing receipt of services or standing in an organisation.

The age of majority varies across countries, and in some, between jurisdictions within the same country. In instances where a parent or guardian is required to give consent for a child’s participation in research, it is still important to obtain assent (i.e. verbal or written agreement to participate) from the child. Specific adaptations may be needed in these procedures for children with disabilities. For example, children with intellectual impairments may require information in simplified formats, while children with visual impairments may require information sheets be written in Braille or read out loud. Further, while written consent is typically preferable, recorded or witnessed oral consent may be more appropriate for children with visual impairments, who are unable to read and/or write or with certain physical impairments.

For children and young people over the age of majority but who have intellectual or other impairments that affect their ability to give informed consent, the decision on when permission on their behalf (i.e. proxy consent) can be sought and by whom is complex and not always clearly defined in local laws and IRB regulations [66]. In some countries, consent may be given by a person’s guardian or an organisation representing their interests (e.g. institutional home) if there is any doubt on a person’s legal capacity. However, the UNCRPD has advocated for ‘supported decision-making’ rather than substituted decision-making. Supported decision-making gives primacy to a person’s will and preferences instead of delegating a person’s decision-making power fully to another [67]. With supported decision-making, planning, advocacy, and communication supports (e.g. eye boards, sign language) are made available to enable a person to advocate on behalf of themselves to the greatest extent possible. Information on a participant’s preferred communication style should be sought in advance of data collection where possible to allow for appropriate planning. Further, participants’ level of understanding should also be verified independently by data collectors, as caregivers may underestimate or misconstrue their child’s abilities.

When available supports are insufficient to determine a person’s view, representational support may be required [67]. Even in this case, the representative should be someone who knows the person with a disability well, and can provide context of the person’s identity, interests and preferences in order to inform decisions made on their behalf. Representatives are ideally selected with input from the participant; in the rare circumstances where this is not possible, a legal guardian or the person most involved in the individual’s daily care and support are likely the most appropriate option. In addition to applying supported-decision making, local laws on legal capacity should be reviewed ahead of research if available.

All uses of a proxy, whether used in lieu or in conjunction with direct feedback from the participant, should be documented during data collection and considered during analysis (e.g. disaggregation of findings by proxy vs non-proxy response).

#### Child protection responses

In order to conduct good quality ethical research on violence, provision must be made to thoroughly address any child protection issues that are identified as a result of the study. This usually necessitates the identification of existing state, civil society, and community resources that might be able to offer good quality referral services to children. Ensuring that community leadership is aware of the study is also an important step when attempting to recruit community resources. Well thought-out child protection systems also involve the development of a clear, written ‘referral plan’ which outlines roles and responsibilities, specifies how violence necessitating intervention will be defined, what support may be provided, and from whom [33]. Some disclosures of violence may lead to discretionary referrals, where participants may choose whether or not to go through with the proposed strategies; however, for certain disclosures (e.g. sexual violence, instances where the individual’s welfare is in immediate danger) and for certain children (e.g. under the age of consent, people with limited legal capacity), or for certain professionals (e.g. doctors, nurses, teachers or social workers), referrals may be mandatory and involve breaking confidentiality [68]. Mandatory reporting requirements may conflict with researchers’ role in data collection and as such, the research team should clearly outline before data collection when mandatory referrals are required. In all referrals, however, it is essential to engage with the child (and his/her guardian if appropriate) and respect their wishes on what they want to happen next as much as possible.

In many contexts, child protection systems are weak, and so the research study will need to develop a referral network which builds on existing structures and services as much as possible. Child protection systems that are accessible to children with disabilities are particularly limited, or non-existent, in many contexts. A concern is that referral services that are only available during the duration of the project raise ethical dilemmas for researchers where children require support after the lifetime of the study, and where local services are not able to sufficiently respond. It is therefore paramount to work with existing systems wherever possible. Poorly handled referrals may also cause harm, emphasising the value of working with partners about whom researchers can be confident can appropriately protect children and address their needs.

Even where child protective services do exist, some children, particularly those with disabilities, face physical, social, economic, and institutional barriers to their access [69], and so plans must be made for how these are overcome. For example, many services are urban-based, which presents challenges for many children living in rural areas, but is particularly difficult for children with mobility limitations when accessible transportation is not available. Furthermore, child protection facilities may not be physically accessible, may not provide information in accessible formats and staff are often not trained to work with children with disabilities, particularly children with communication or intellectual difficulties. Children with communication difficulties may experience difficulties sharing their experiences and what they want to happen next without appropriate accommodations. The research team should therefore consider not just the availability of referral services, but also their accessibility to children with diverse disabilities and what, if any, supports the research team and its partners can provide to overcome gaps in formal services.

Children with disabilities may also require additional referral pathways, such as to health and rehabilitation services (e.g. provision of specific assistive devices) and to disability-targeted programmes (e.g. social protection, inclusive education, caregiver support groups). Researchers therefore need to be aware of which facilities are available, and have prior agreement with these providers that they are willing to accept referrals of children with disabilities identified through the research. Working together with local DPOs and/or Community Based Rehabilitation programmes may help to identify the relevant services, and establish networks for referrals.

Researchers may also have to consider whether proceeding with the research is ethical if services are not available and an appropriate referral plan cannot be developed. This decision has to be weighed against the risks of not conducting research on this sensitive topic if this research has the potential to improve policy or practice and positively impact children’s lives.

### Collecting data about violence among children with disabilities

#### Consulting with children with disabilities

Children and young people should be consulted and included in any research, intervention, programme, activity or policy which has the potential to impact their lives. This consultation can help to define the aim of the research, the methods used (e.g. how the population is sampled, which topics are covered, how data is collected), how the results are analysed and interpreted, and consideration of the strategies for dissemination of research findings.

Different approaches may be needed when consulting with children and young people with disabilities, depending on the nature of the impairment underlying the disability (Table 1). Participatory approaches may for example enable children with disabilities to contribute in a way in which they are most comfortable. While this may make it more difficult to design methods that best accommodate different children’s specific needs, all approaches should build on what a child is *able to do* in order for appropriate adaptations to be made if necessary. Sufficient time, resources and budget must be allocated to promote approaches that maximise children’s inclusion. For instance, sign language interpretation is important for communicating with a child with a hearing impairment (if the child knows formal sign language), while the use of simplified language is helpful for consulting with a child with an intellectual impairment. It is important to consult with the child and/or caregiver before the interview to work out which kinds of adaptations may be needed during the interview. Local DPOs may also be useful sources of information and support. The changes in interview approach required for children with disabilities are often small and easy to achieve with limited budget implications. Furthermore, learning skills to consult effectively with children with different impairments will help interviewers to communicate better with children in general, regardless of whether they have a disability or not.

**Table 1:** Considerations when consulting with children with specific impairments [70]

Impairment type	Considerations during interview
Hearing impairment	• Be still and face the person at all times when speaking. • Speak slowly and clearly, with simple language and using a steady rhythm. • Keep background noise to a minimum. • Get the child’s attention before you start speaking, using visual cues (e.g. wave or tap the participant on the shoulder). • If you think you have not been understood, do not repeat the sentence. Think of ways to rephrase your sentence. • If an interpreter is present, still speak directly to the child. • Provide notepads for children who can read/write. • Use written explanations, diagrams/pictures to emphasise points.
Physical impairment	• Never address the escort or personal assistant instead of the person with a disability, always talk to the person directly. • If you are talking to someone in a wheelchair or who is sitting down, ensure you sit at the same level so you are face-to-face. • Wheelchairs and other assistive devices are very much part of their personal space - do not lean or sit on it. • Respect a person’s independence and don’t make assumptions about what he/she can or cannot do. • Ensure meetings take place in accessible environments.
Visual impairment	• When you approach a person who is blind remember to identify yourself clearly, and tell them who else is present. • Tell them when you are leaving or moving away - do not leave someone talking to an empty space. • Always face the person when talking with them. • Use their name to get their attention. • Keep background noise to a minimum. • Give a clear verbal description of the surroundings and any visual information you are using. • When leading, offer your elbow, wait for their consent, and walk slightly ahead.
Intellectual impairment	• Give clear, concise instructions to the child. • Be prepared to explain more than once, if the person does not understand the first time. • Be patient and give positive reinforcement, but don’t put ‘words into their mouths’. • As much as possible, communicate directly with the person. If he/she has challenges communicating independently, suggest involving a caregiver or other suitable person. • Many people – including caregivers – underestimate the abilities of people with intellectual impairments. Interact with the child or young person yourself; get their feedback and don’t assume direct consultation is not possible.
Communication impairment	• Be encouraging and patient. Don’t speak for the person and do not correct them. Wait until the person finishes and resist the temptation to finish sentences for them. • Where possible, ask questions that require short answers or just a nod or shake of the head. • If you do not understand, do not pretend that you do. Repeat as much as you did understand and use the person’s reactions to guide you. Ask them to tell you again, if necessary. • Even when using adaptations, information gathered is often limited. Seeking additional details (when appropriate) from a friend/guardian for context can be helpful.

It is worth noting that many accommodations and adaptations can be provided at low or no cost, particularly if disability is considered early in the development of research plans. For example, large text/recorded/simplified/read-out versions of materials carry little cost. Participants and their friends and family may also have developed unique methods for communicating that can be drawn on (e.g. informal sign language, picture-based communication). Other accommodations may require appropriate budgeting, such as for sign-language interpretation or for people trained in working with children with intellectual or communication impairments. In some contexts, the authors have included knowledge of sign language and experience with working with children with disabilities as desirable characteristics when recruiting data collectors, which both improves the quality of data collection and can reduce costs of hiring additional staff.

As much as possible, children should be interviewed directly and in privacy. Input from caregivers on how best to communicate with their children is important; however, as highlighted in the section on consent, interviewers should also independently verify the child’s communication abilities as caregivers may underestimate or misconstrue a child’s abilities. Involvement of caregivers in interviews should be limited (unless requested by the child) and used only after exhausting a range of communication strategies, particularly if exploring violence within the home. Any involvement of a caregiver during data collection (e.g. as a proxy, as a communication aid or even if they are present during the interview) should be documented and considered during analysis.

The consultation process will help to refine the approaches of the research, and the following areas will need to be considered (as discussed below): sampling the population, data collection, analysis of results and dissemination of findings.

#### Sample selection of children with disabilities

Ensuring the experiences of children with disabilities are adequately represented within a study requires some additional considerations when establishing methods for sampling. Different sampling methods have different opportunities and constraints. Schools are often used as sites of recruitment for studies of children because they are a convenient way of identifying large numbers of children. However, as children with disabilities are much less likely to be in school than their peers without disabilities [27] school-based surveys risk under-representing the experience of children with disabilities. Children with intellectual and communication impairments have particularly low rates of school enrolment [27], resulting in their views being most likely to be excluded with school-based recruitment. Further, appropriate measures are needed with school-based recruitment to ensure confidentiality and a clear distinction between research and school processes, as violence against children with disabilities in schools is common and can be perpetrated by peers and school staff alike [30, 69, 71]. Furthermore, children in schools can also be described as a ‘captive sample’ to the extent that they may not be able to opt out of an activity if for example school norms, rules and conventions make it difficult for them to decline to participate. This may be further exacerbated where there aren’t any options, for example separate activities, rooms or supervision for students who choose not to participate in research activities. Such challenges mean that recruitment may therefore usefully include community settings for studies focussing on violence among children with disabilities.

It may also be warranted to specifically target children with disabilities for inclusion in studies in order to achieve a sufficient sample size, for instance through collaboration with Disabled People’s Organizations (DPOs). However, children who are known by DPOs may not be representative of the broader population of children with disabilities. For example, these children may live closer to the DPOs office (which tend to be in urban centres), have more “recognisable” disabilities (e.g. physical impairments as opposed to mental health conditions) or may have caregivers who are more well-connected, knowledgeable and accepting of disability and aware of relevant services. Furthermore, children known to DPOs may face less social exclusion than other children with disabilities, who may be more physically, economically or socially marginalised. It is therefore important to understand and evaluate potential biases that may arise when identifying children with disabilities using these strategies in order to appropriately interpret study findings.

As with all research, population-based recruitment is most representative and least prone to bias; however, large sample sizes are required to identify sufficient numbers of children with disabilities to then explore violence (given a 5% prevalence of childhood disability and 25% prevalence of violence amongst children with disabilities [19]). However, disability is increasingly being measured in censuses and other large-scale surveys (e.g. UNICEF’s Multiple Indicator Cluster Surveys), which presents opportunities for gathering population-based data on violence in children with disabilities.

Another important consideration is to ensure that study methods do not result in an unintentional exclusion of children with disabilities from the sample. For example, written surveys may be inaccessible to children with visual or intellectual impairments, while requiring participants to travel to an interview site may limit participation for children with physical impairments. Directly interviewing children with intellectual or profound hearing impairments may require alternative communication strategies (e.g. simplified language, pictorial representations, sign language). Online data collection tools should also ensure their platforms are accessible (e.g. content can be used by screen-readers, include large-text or simplified formats), while also considering the need for other data collection avenues given that children with disabilities, particularly in LMIC contexts, may not have access to the internet or assistive technology.

### Data collection: interviewer considerations

Selecting and training interviewers who are sensitive to the needs of children, including children with disabilities, and who have experience of working with vulnerable groups, is important for ensuring good quality, ethical research [33].

#### Interviewer selection

Bearing local cultural norms in mind, researchers should consider whether interviewers should be gender-matched to interviewees given that the gender of the interviewer can impact on the openness of respondents and thus the quality of data [38]. Interviewers should also be fully aware of local cultural and contextual realities and fluent in the language spoken by children (including sign language if possible), so that children are given the opportunity to communicate in the language in which they are most comfortable. To the extent possible, the research team would ideally also include people with disabilities. The involvement of people with disabilities as interviewers may help to develop rapport with study participants with disabilities, and allow for a better exploration of sensitive topics, such as stigma and discrimination of disability. Furthermore, while needing to be empathetic to children’s experiences, the role of the interviewer must not extend to counselling. This is a specialised skill and without appropriate training, interviewers risk providing inadequate or inappropriate support or themselves being overburdened by the attempt to counsel. This concern further reiterates the importance of an appropriate referral system in order to respond appropriately to the needs of research participants.

### Interviewer training

Training interviewers on study tools, procedures and techniques, and the assessment of both violence and disability is also important not just for the fidelity of the data, but also to ensure that research and child protection procedures and protocols are strictly adhered to [33, 72]. Training should include opportunities to practice key research skills including building rapport, maintaining confidentiality, active, non-judgemental listening, and identifying distress [33].

Training interviewers to conduct good quality, ethical data collection with children with disabilities should include specific training and sensitisation on issues related to violence and disability. Such efforts can be supported through the involvement of DPOs who may be able to assist with specific guidelines to help children with disabilities participate as fully as possible. Impairment specific challenges in working with children with disabilities mean that interviewers will often require specific guidance on how to communicate as effectively as possible with children, while emphasising and building on what children are able to do (See Table 1) [73]. Inherent difficulties in obtaining complete or candid disclosure from children in households where household members perpetrate violence against a child must however be acknowledged, particularly where children have communication impairments and rely on household members to help them communicate. An important aspect of the training is also ensuring that interviewers are fully supported by senior research team members to appropriately refer any children who are identified through the referral pathway or as part of their participation in the study, to appropriate services. This may also include reporting child maltreatment or abuse to appropriate authorities. Research participants should also be made aware that interviewers may therefore break confidentiality in instances where there are concerns for the welfare of the child [68].

### Interviewer welfare

Research can be emotionally draining for interviewers, making it necessary to consider both the physical and emotional risks to which interviewers may be exposed [74–77]. These are important considerations as they may affect interviewers’ ability to respond appropriately to children and protect their wellbeing, the quality of the data collected, and the integrity of sampling procedures (e.g. avoidance of interviewing children who they suspect may disclose information that they will find distressing). In order to conduct ethical research, interviewer welfare should be carefully considered through the development of an effective interviewer care plan. Here, effort is required at a number of different levels. During study preparation and planning, time and financial resources should be budgeted to ensure that data collection targets can be met whilst allowing time for interviewers to reflect on their experiences and share any challenges they may be experiencing (e.g. by scheduling regular team debriefs). Interviewers should also have access to referral or support services, should they need them. The research management team should also be approachable and genuine in their efforts to ensure interviewer welfare so that interviewers feel valued and supported. The training offered to interviewers is also very important, particularly for interviewers who are new to researching difficult or sensitive topics such as violence or the experience of children with disabilities. A responsible research care plan should also consider the needs of research supervisors who in turn are often the first line of support to interviewers and may be the first to detect signs of interviewer stress (e.g. reduced performance, apathy, change in personality). As with study participants, it is also important for research management to consider where their responsibility to the welfare of interviewers ends following the completion of data collection.

### Data analysis and the importance of dissemination of results and influencing practice

While improving the measurement of violence against children with disabilities through well designed and ethical research is important to better understand the prevalence of violence, so too is the analysis and dissemination of the results. Opportunities should be created to include children with disabilities in data analysis activities or as part of participant checking of preliminary findings to ensure that the results adequately reflect their views and experiences. This offers important opportunities to design better policies and interventions to meet children’s needs as well as respond to the violence that they may be experiencing. As with the research, any efforts to disseminate research findings must also be sensitive to the impairments that children may have, such that information is disseminated in a way that is accessible to them. This could for example involve making a Braille or audio version of appropriate dissemination materials available to children with visual impairments. Dissemination should also extend from local to national and international levels so that the findings of the research may contribute to improving the lives of children with disabilities in varied settings. As much as possible children with disabilities and DPOs should also be meaningful involved in the dissemination of research findings.

### Case study of the good school study Uganda

Bringing all these factors together requires careful consideration and planning. Table 2 summarises the experience of the Good School Study which was conducted in a district in Uganda and sought to evaluate the impact of the Good School Toolkit, an effective whole school intervention that successfully reduced past week violence from teachers to students in intervention schools as compared to control schools (odds ratio 0·40, 95% CI 0·26–0·64, *p* < 0·0001) through improving student-teacher relationships, clarifying student behaviour expectations through rewards and praise, and encouraging positive discipline and alternative discipline methods [78, 79].

**Table 2:** The good school study

Study Aim	To evaluate the Good School Toolkit – a whole school intervention designed by Raising Voices (www.raisingvoices.org) which seeks to reduce violence in schools.
Methodology	Multi-component study (randomised controlled trial, qualitative evaluation, process evaluation, economic evaluation) conducted between 2012 and 2014. Interviewed students, teachers, school staff, school administration, parents/carers.
Study Measures	Quantitatively violence was measured trough self-reports using the International Society for the Prevention of Child Abuse and Neglect (ISPCAN) Child Abuse Screening Tool – Child Institutional (ICAST-CI). Disability was measured using the Washington Group Short Set Questions. Qualitatively in-depth interviews using a semi-structured tool were used to explore children’s subjective experiences and understanding of violence and how it impacted on their lives. Interviews with children with disabilities further explored how their disability affected their experience of school and their ability to participate in the intervention.
Ethical Approval	Obtained from the London School of Hygiene and Tropical Medicine and the Uganda National Council for Science and Technology
Permission	Obtained from appropriate national and district government authorities
Consenting Procedures	3-tiered consent process: 1. Consent sought from head teachers for school participation and to approach parents and students 2. Parents given opportunity to opt their children out of participation 3. Sampled children asked to given written consent to participate after emphasising that participation was voluntary, that there existed an obligation to report in case of any disclosure of violence, and that referral services were available. Specific attention was given to ensuring that children with disabilities understood the information with which they were presented and consented to take part by ensuring that information was provided in a manner that was accessible to them for example slowly and clearly for children with auditory impairments.
Interviewer recruitment and training	All interviewers had previous experience of working with children and provided written references. They received 3 weeks of full-time training on violence against children, child rights, disability, strategies to maintain privacy and confidentiality, consenting procedures, interview techniques and referral protocols. Considerable time was also made available for role-playing and practicing to ensure interviewers were as prepared as possible e.g. to respond appropriately to disclosures of violence. Interviewers were also trained on specific techniques for interviewing children with disabilities for example ensuring that they talked slowly and clearly whilst also facing children with auditory impairments. These techniques were practiced through role playing. An excess of interviewers were trained with only the best invited to participate in the data collection.
Referral procedures	The study employed a counsellor. A comprehensive protocol to handle disclosures of violence was developed in consultation with local child protection experts. It specified specific pathways of action depending on the severity and timeframe of what the child disclosed. Decisions on disclosures that would necessitate a referral and to where they would be referred were made considering the legal requirements in Uganda and the local child protection systems.
Interviewer welfare	The study measured signs of vicarious trauma amongst interviewers and provided space for critical reflection through team debriefs and space to decompress through group social activities. Interviewers were also able to speak with the study counsellor.
Challenges and learning	At follow-up, training on disabilities, specifically epilepsy (which is almost always considered a disability in this context), was strengthened given that at baseline, some interviewers were found to have a poor understanding of this disability. Children with the most severe disabilities were unlikely to be at school meaning that children with disabilities who were included in the study are likely to have less severe impairments.

### Methodological gaps in the conduct of research on violence against children with disabilities

While with careful consideration and planning, much can be done to improve the quality of inclusive research on violence against children, important methodological gaps remain. The first relates to how to include children with severe communication challenges (e.g. children with severe intellectual impairments or children with profound hearing impairments but no sign language skills), which make it difficult for them to share their views and experiences. These children may arguably be at extremely high risk of violence, especially where they are unable to disclose their experiences and thus potentially access protection or the support of others to advocate on their behalf. This challenge may be most acute in qualitative research meaning that the subjective experiences of violence of children with communication challenges may be especially under represented.

Particularly in contexts with limited child protection provisions, another methodological challenge arises around knowing how best to respond when a parent or carer is the perpetrator of violence. In some instances, a child may be sent to another member of the extended family to be cared for, but this is not always the case. Knowing how best to protect a child who is experiencing violence at the hands of a parent or guardian when appropriate alternative care options are limited, highlights the limitations not only of child protection services, but also the referral options within a study on violence against children, particularly as poorly handled referrals may cause additional harm.

Methodological uncertainty also exists in determining how best to design sampling procedures to ensure that surveys adequately represent children with disabilities in large-scale violence prevalence surveys. As noted above, children with disabilities are less likely to attend schools than their counterparts without disabilities making school-based prevalence assessments likely to underreport the experience of children with disabilities. While community or household-based surveys may offer more opportunity to identify children with disabilities, they may also be affected by stigma and discrimination that often reduces the participation and visibility of children with disability in their communities. Similarly, methodological and ethical questions also arise in determining how best to ask safely about violence in large scale surveys and in on-going monitoring data, for example that routinely collected by schools. This will require appropriate training of interviewers and also ensuring that effective child protection and referral services are available for any children that have been identified to be at risk of violence, which may also be beyond the capacity of many of these surveys.

## Conclusions

Childhood disability is common, and children with disabilities are known to be particularly vulnerable to violence. There are important gaps in our knowledge of why this vulnerability exists, and how best to prevent and respond to violence perpetrated against children with disabilities. More research is therefore needed on this topic. There are also key methodological gaps remaining that challenge the investigation of violence against children with disabilities. As researchers, IRBs, child protection systems, funders, and service providers work to overcome these methodological challenges, what high quality, ethical and inclusive research on violence against children that can be safely done should be encouraged, so that evidence can be collected on this neglected area.

## Data Availability

Not applicable

## Abbreviations

DPO: Disabled Persons’ Organisations
HBSC: Health Behaviour in School-Aged Children
ICAST: ISPCAN Child Abuse Screening Tools
IRB: Institutional Review Board
ISPCAN: International Society for the Prevention of Child Abuse and Neglect
JVQ: Juvenile Victimisation Questionnaire
LMIC: Low and Middle-Income Countries
MICS: Multiple Indictor Cluster Surveys
SDGs: Sustainable Development Goals
UNCRPD: United Nations Convention on the Rights of Persons with Disabilities
VAC: Violence Against Children
VACS: Violence Against Children Surveys
WHO: World Health Organization
